# Persistence of Anti-Rabies Antibody Response in Horses Following Vaccination

**DOI:** 10.3390/pathogens13020125

**Published:** 2024-01-28

**Authors:** Sharon Tirosh-Levy, Leehe Shaiman Barom, Shiri Novak, Marina Eyngor, Gili Schvartz, Boris Yakobson, Amir Steinman

**Affiliations:** 1 Koret School of Veterinary Medicine, The Robert H. Smith Faculty of Agriculture, Food and Environment, The Hebrew University of Jerusalem, Rehovot 7610001, Israel; leehe.shaiman@mail.huji.ac.il (L.S.B.); giliun@gmail.com (G.S.); amirst@savion.huji.ac.il (A.S.); 2 Division of Parasitology, Kimron Veterinary Institute, Beit Dagan 5020001, Israel; 3 WOAH Rabies Reference Laboratory, Kimron Veterinary Institute, Beit Dagan 5020001, Israel; shiri.novak@mail.huji.ac.il (S.N.); marinal@moag.gov.il (M.E.); boris.yakobson@gmail.com (B.Y.); 4 Department of Virology, Kimron Veterinary Institute, Beit Dagan 5020001, Israel

**Keywords:** equine, horse, immunity, rabies, RVNA, vaccine

## Abstract

Rabies is a fatal zoonotic disease affecting all mammalian species. It is caused by the rabies virus and is prevalent worldwide. Horses are not commonly infected with rabies but their vaccination is recommended due to the potential zoonotic risk. This study aimed to evaluate the duration of immunity following rabies vaccination in horses. A total of 126 serum samples were collected from 93 horses, vaccinated 6 to 91 months before sampling. Rabies-virus-neutralizing antibody (RVNA) levels were evaluated using the Rabies Fluorescent Focus Inhibition Test (RFFIT). A protective RVNA titer of above 0.5 IU/mL was found in 112 (88.9%) of the samples and 84 (90.3%) of the horses. Antibody titers declined over time (rho = −0.271, *p* = 0.002); however, there was no significant difference in antibody titers or the prevalence of unprotected horses between the time intervals following vaccination. Purebred horses had lower antibody titers (*p* = 0.024). The response to booster vaccination was inspected in ten horses, and increased antibody titers were found in eight of them. The results of this study demonstrate the prolonged persistence of protective immunity in horses following rabies vaccination, in some cases, for up to eight years. Therefore, the current annual vaccination strategy should be re-evaluated. A rate of 9.7% of poor responders should be considered from an epidemiological perspective in order to minimize the risk of emergence of the disease.

## 1. Introduction

Rabies is a fatal zoonotic disease caused by the rabies virus, a negative-sense single-stranded RNA virus of the *Lyssavirus* genus, the *Rhabdoviridae* family and *Mononegavirale* order [1]. Rabies is present throughout the world (except Antarctica) and leads to tens of thousands of human deaths every year, mostly in Asia and Africa. Although the main source of infection in humans is dogs, rabies can affect all mammalian species, wild and domestic. In all affected species, clinical disease involves the central nervous system and, once clinical signs appear, there is no effective treatment and the outcome is nearly always fatal [1,2].

Rabies is preventable by vaccination, and all prevention, control and treatment programs are based on vaccination [2,3,4,5]. Pre-exposure prophylaxis vaccines are given to people who are considered at high risk of exposure (veterinarians, laboratory workers, etc.) to domestic animals, in order to reduce exposure and spread to humans (mostly dogs and animals in close contact with humans, such as in petting zoos), and to wild animals (mostly wild canids) via baits, in order to reduce the risk of infecting domestic animals or humans. In many countries, rabies control programs include mandatory vaccination of pet dogs and animals at petting zoos in combination with bait-vaccination of wild animals. Post-exposure prophylaxis is administered to people after exposure or suspected exposure to rabies in order to prevent clinical disease [2,3,4,5].

Most vaccination protocols for pre-exposure prophylaxis in humans and pets involve primary vaccination of three injections, followed by an annual booster vaccination. In humans, it has been shown that immunity following the first one-year booster vaccination was maintained, in most cases, for ten years [6,7,8]. Therefore, the suggested protocol for immunized people is to periodically measure their antibody titer and revaccinate only if their antibody titers are below the established protective titer of 0.5 IU/mL [9]. Long-term immunity following vaccination has also been demonstrated in cats and dogs, with sufficient immunization established when a booster is administered every three years [10,11].

Rabies is not common in horses, and transmission from horses to humans is rare [12]. Nevertheless, due to the fatality of the disease to horses and its zoonotic risk in animals in close contact with humans, vaccination of horses against rabies is advisable in endemic areas. The recommended protocols for rabies vaccination in horses usually suggest annual immunization [13]. Few studies have investigated immunity in horses following rabies vaccination, and these have mostly focused on the effect of age on the efficacy of vaccination [14,15]. However, as in other animals, prolonged immunity has been suggested in horses of all ages for up to at least three years [16].

Rabies is endemic in Israel, with dozens of confirmed cases in animals annually. The majority of cases are in canids (jackals, dogs, wolves and foxes), with spillover to domestic herbivores, including cattle, small ruminants and horses. Only five (0.98%) of 509 confirmed cases since 2010 were in horses [17]. Israel has an extensive control program against rabies that includes mandatory annual vaccination of all domestic dogs, the spread of oral vaccines for wild canids, and the requirement for annual vaccination of all animals in close contact with humans (such as those in petting zoos, riding schools, equine events, etc.). However, vaccination is not mandatory for privately owned animals other than dogs. Currently, most horse owners and breeders in Israel choose to vaccinate against rabies annually, as recommended by most equine practitioners.

This study aimed to evaluate the duration of protective immunity (above 0.5 IU/mL) in horses following vaccination by measuring rabies-virus-neutralizing antibody (RVNA) levels in horses at different time intervals since their last vaccination. In addition, the antibody response following booster vaccination was evaluated in a small subset of horses in order to evaluate the effect of the annual revaccination regime. 

## 2. Materials and Methods

### 2.1. Study Design

This study was designed as a retrospective study aimed at measuring the levels of rabies-virus-neutralizing antibodies (RVNA) in horses at different times since their most recent vaccination. The samples analyzed were serum samples collected from horses at different farms throughout Israel as a part of surveillance studies during 2015–2018. Originally, we aimed to test 20 horses at each of the following intervals since vaccination: six months, one year, eighteen months, and two, three, four, five, six, seven and eight years. However, since most horses in Israel receive annual vaccination, it was difficult to recruit enough horses that had not been vaccinated for over two years and that had information regarding the date of their last vaccination. Therefore, we actively resampled 33 horses during 2020–2021 that were not vaccinated during the study period in order to increase the number of horses in the groups with larger intervals since vaccination. 

Eventually, 126 samples were included in the analysis, which were divided into four categories: (1) 76 samples collected between six months and two years following vaccination, (2) 21 samples collected over two years and up to four years following vaccination, (3) 19 samples collected over four years and up to six years following vaccination, and (4) ten samples collected over six years and up to eight years following vaccination.

In addition, serum samples were collected from ten horses immediately before and a week after administration of their annual booster vaccination.

### 2.2. Study Population and Sample Collection

The serum samples included in this study were collected from 93 horses at 11 farms in Israel. All horses were apparently healthy during sampling. Blood was collected from the jugular vein of each horse into sterile serum collection tubes without anticoagulating agent. Serum was separated by centrifugation at 2500 rpm for six minutes and stored at −20 °C until testing. Data regarding each horse’s age, sex, breed, housing management and date of last anti-rabies vaccination were collected from the owners upon sampling.

Sample collection was performed under the owners’ consent. The samples collected during the 2015–2018 surveys were approved by the Koret School of Veterinary Medicine, Veterinary Teaching Hospital Internal Ethics Research Committee, approval numbers KSVM-VTH/23_2014 and KSVM-VTH/02_2018, and the additional samples collected during 2020–2021 were approved by the Hebrew University Ethical Committee, approval number NER-2020-055-A.

All horses that were included in this study had received at least one dose of anti-rabies killed vaccine (RABISIN^®^, Boehringer Ingelheim, Ingelheim, Germany) administered intramuscularly prior to sampling.

### 2.3. Measuring Rabies-Virus-Neutralizing Antibody Levels (RVNA)

Samples were tested for rabies antibodies at the Kimron Veterinary Institute serving also as WOAH rabies reference laboratory, using the Rabies Fluorescent Focus Inhibition Test (RFFIT) [18]. The test is ISO 17025 accredited by The Israel Laboratory Accreditation Authority. Mouse neuroblastoma cell line (MNA, Wistar Institute, CDC, Philadelphia, PA, USA) was used for culture. The challenge virus used was challenge virus standard-11 (CVS-11, Nancy, France). Secondary antibodies used were FITC anti-rabies monoclonal globulin (Fujirebio Diagnostic Inc., Malvern, PA, USA). Samples were diluted at a 1:10 ratio, starting with undiluted serum and ending at 10^−3^. Positive and negative control samples were added at each session. RVNA levels were calculated according to interpretation of the fluorescence results [18].

### 2.4. Statistical Analysis

The correlation between RVNA and the time elapsed since last vaccination was calculated using Spearman’s rho correlation coefficient. The associations between RVNA and the different time intervals and other potential risk factors were evaluated using Kruskal–Wallis nonparametric test (as RVNA did not have normal distribution). All factors, which were significantly associated with RVNA in the univariable analysis, were included in a multivariable generalized estimating equation (GEE) model with logit link function, with the horse defined as subject and the farm as within subject effect. The difference between RVNA levels at different time points in horses that were sampled twice was assessed using related-samples Wilcoxon signed rank test. Statistical significance was set at *p* < 0.05. The analysis was performed using SPSS 25.0^®^ and Win Pepi 11.43^®^ statistical software.

## 3. Results

### 3.1. Study Population

A total of 126 samples were collected from 93 horses at 11 farms, between two and 29 at each farm. Thirty-three horses were sampled twice at different times following vaccination. The majority of samples, 63.5% (80 of 126 samples), were collected from mares, 31.7% (40 of 126) from geldings and 4.8% (6 of 126) from stallions. Forty samples (31.7%) were collected from horses of mixed breeds, 30.2% (38 of 126) from Tennessee walking horses, 23% (29 of 126) from Arabians, 9.5% (12 of 126) from Quarter horses, 4.8% (6 of 126) from Warmbloods and one sample (0.8%) from a miniature horse. The age of the horses during sampling ranged between two and 38 years (mean = 12.1 years, standard deviation (SDV) = 5.66, median = 11.25, interquartile rang (IQR) = 7.3). Information regarding the housing management was available for 117 of the samples, of which 29.9% were collected from horses kept in stalls (35 of 117 samples), while 70.1% were from horses kept partially or fully in paddocks (82 of 117 samples).

**Figure 1:**
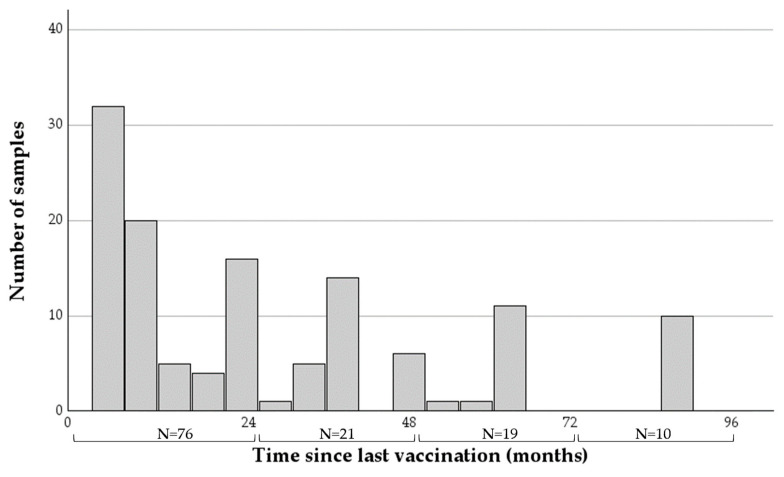
A histogram describing the time intervals between the last rabies vaccination and sample collection of the 126 samples collected from horses analyzed in this study. The number of horses at each two-year category appears at the bottom.

The time intervals from last vaccination to sample collection ranged between six and 91 months (mean = 30.42, SDV = 25.76, Figure 1), with the majority of samples (76 of 126) taken within two years of vaccination.

### 3.2. Changes in RVNA Levels over Time Following Vaccination

**Figure 2:**
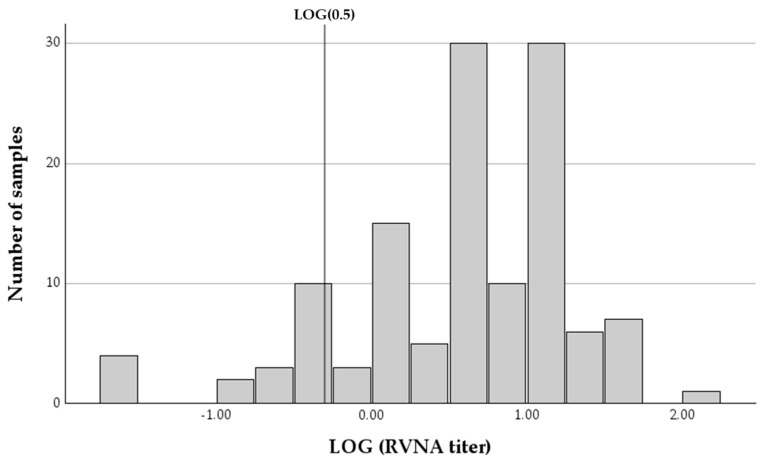
A histogram of the logarithmic distribution of rabies-virus-neutralizing antibody titers (RVNA) in 126 samples collected from horses at different time intervals since last vaccination. The vertical line represents the LOG of the protective threshold of 0.5 IU/mL.

RVNA end point titers of all samples ranged between 0.03 and 104.14 IU/mL, with a mean of 9.39 IU/mL (SDV = 13.192, 95% confidence interval (CI): 7.06–11.71) and a median of 4.7 IU/mL (IQR = 11.2). The vast majority of samples (112 of 126, 88.9%, 95% CI: 82.1–93.8%) had antibody titers above the established threshold for protection against infection (0.5 IU/mL), with most horses (113 of 126, 89.7%, 95% CI: 83.0–94.4%) having antibody titers below 20 IU/mL. Only 14 of the samples (11.11%, 95% CI: 6.2–17.9%) had antibody titers below the protective threshold (Figure 2). Since some of the horses were sampled on two occasions, in some cases, horses were below the protective titer on two occasions. A total of nine of 93 horses (9.7%, 95% CI: 4.5–17.6%) had RVNA titers below the protective cutoff on the first sampling date. 

A weak yet significant negative correlation was found between the time elapsed since vaccination and RVNA titers (rho = −0.271, *p* = 0.002), which indicates a slight decline in antibody levels over time. However, no significant difference was found between the distribution of RVNA titers in the different time intervals since last vaccination (Kruskal–Wallis *p* = 0.227). Moreover, no significant difference was found in the proportion of horses with antibody titers below the protective threshold at different time intervals post-vaccination (Table 1).

### 3.3. Potential Risk Factors Which May Be Associated with RVNA Titers

Although the time since last vaccination was not found to be significantly associated with antibody titers, the risk factor analysis was performed only on the samples that were collected within two years of vaccination (N = 76). The horses in this group originated from ten farms, 46% of them were of mixed breeds, while the rest were of various breeds (Table 2), and horses’ ages ranged between two and 38 years (mean = 11.27, SDV = 5.98).

In the univariable analysis, the farm (*p* = 0.001), horses’ breed (*p* < 0.001), sex (*p* = 0.012) and age (rho = 0.410, *p* < 0.001) were found to be significantly associated with RVNA titer (Table 2). When exploring possible interactions between variables, the farm was significantly associated with horses’ breed, sex and housing management (all *p* < 0.001); therefore, the farm was included as “within subject effect” in the multivariable model. The horses’ breed (*p* = 0.002), sex (*p* = 0.817) and age (*p* = 0.110) were included in the multivariable model, with only breed remaining significant. Tennessee walking horses, warmbloods and miniature horses had significantly lower antibody titers than mixed-breed horses (Table 2). This association remained significant also when horses’ breed was analyzed as a dichotomous parameter, with purebred horses having significantly lower antibody titers than mixed-breed horses (*p* = 0.024).

Horses’ breed (*p* = 0.702), sex (*p* = 0.313), age (*p* = 0.114), farm (*p* = 0.685) and housing management (*p* = 0.667) did not significantly associate with the risk of not having a protective antibody titer (<0.5 IU/mL). 

### 3.4. Repeated Samples of Individual Horses

Thirty-three of the horses were sampled on more than one occasion following vaccination, without receiving an additional booster vaccine between sampling dates. Horses were revisited at four of the farms and at different times since last vaccination, as specified in Table 3. 

Of these 33 horses, 17 demonstrated a decrease in RVNA titer between samplings, while 16 had an increase in antibody titer. Antibody titers at the first sampling ranged between 0.16 and 48.63 IU/mL (mean = 7.89, SDV = 12.01, 95% CI: 3.64–12.16), while, in the second sampling, it ranged between 0.2 and 41.68 (mean = 6.57, SDV = 8.84, 95% CI: 3.44–9.71). There was no significant difference between the medians of RVNA in the two sampling dates (*p* = 0.497). Five of the horses from two of the farms (farms 3 and 4) had RVNA below the protective titer (<0.5 IU/mL), three of which did not have a protective titer at the first sampling either.

### 3.5. Response to Booster Vaccination

Ten adult horses from two farms, aged 3 to 15 years, were vaccinated on the same date after collection of the first serum sample and resampled a week later in order to evaluate the effect of booster vaccination on RVNA levels. The characteristics of each horse and RVNA before and after vaccination are specified in Table 4. The horses marked as Y in the table received their previous vaccination 12 months prior to the first sampling and vaccination, and the horses marked as EH received their previous vaccination 18 months prior to the first sampling and vaccination.

RVNA titers ranged between 1.3 and 14 IU/mL (mean = 6.01, SDV = 4.08, 95% CI: 3.08–8.93) prior to vaccination and between 1.3 and 134.49 (mean = 50.93, SDV = 59.25, 95% CI: 8.54–93.32) following vaccination. RVNA medians were significantly higher following booster vaccination (*p* = 0.011). The magnitude of elevation in antibody titers varied between horses, while two of the horses did not show any increase in RVNA levels following booster vaccination. All horses had antibody levels which provide protection against infection (>0.5 IU/mL) both before and after booster vaccination (Table 4).

## 4. Discussion

The results of this study demonstrate that over 90% of horses maintain their protective immunity against rabies for more than one year and 70% for up to eight years following vaccination. These results support the hypothesis that horses remain immunized longer than the recommended one-year booster interval. Our findings concur with the documented prolonged immunity in pets and in humans [6,10,11,19]. While in humans a ten-year follow-up study was conducted, which proved the persistence of immunity and led to changes in booster vaccination policy, the few studies investigating the matter in pets and horses only performed follow-up of up to three years and immunity was maintained throughout the study period in all animal species tested [10,11,16]. The results of the current study are the first to reveal that immunity may be maintained in horses for a longer period but is based on a small number of horses. Although antibody levels did decrease over time following vaccination, no significant difference in immune protection was found between time intervals since vaccination, and most horses maintained their antibody levels above the protective threshold.

A total of nine of the 93 horses (9.7%, 95% CI: 4.5–17.6%) included in this study had antibody levels below the 0.5 IU/mL protective titer. These horses were tested at different times since their last vaccination and the low titers may have resulted from the gradual decline in RVNA levels after vaccination. However, there was no significant difference in the proportion of unprotected horses between time intervals following vaccination. Moreover, of the 33 horses that were sampled on two occasions, only two dropped beneath the protective level between samplings, while three had RVNA below 0.5 IU/mL on both occasions. These horses may be considered as poor responders to vaccination. Nevertheless, only antibody levels were assessed in this study; this only reflects humoral immune response. Cellular immunity is also a crucial component for protection against viral infection; however, it is not routinely measured in clinical practice or research. Several studies demonstrated that vaccination leads to the development of both cellular and humoral immune responses; however, individual variations in these responses have been demonstrated and, currently, there are not sufficient data to determine if the two correlate and if antibody levels also reflect the extent of cellular response [20]. Variation in antibody levels in response to rabies vaccination between individuals has been described in humans [6], dogs [21,22], cats [22] and horses [16]. Although the design of this study does not allow discrimination between horses with waning immunity and poor responders, the rate of 9.7% poor responders fits the findings of previous studies in horses (4.2%, [16]), dogs (1–20%, [21,22]), cats (2.9%, [22]) and humans (3%, [7]). This rate of poor responders may be significant at the individual level; however, a rate of less than 10% probably does not have much influence at the population level and in the epidemiology of rabies.

Similarly, to humans and other animals, considerable variation in RVNA levels was observed in horses, with antibody titers ranging between 0.03 and 104.14 IU/mL. This range coincides with the previous description of RFFIT results in horses [16]. It appears that the effect of vaccination differs between individuals and the existence and duration of immunity varies accordingly. It had been previously demonstrated that individuals with lower antibody response following vaccination tend to also have poor response to booster vaccination [7,16]. The results of this study support this observation, both by examining the horses with repeated samples and by looking at the response to booster vaccination in the sample of ten horses. Although all ten had protective antibody titers before and after vaccination, the magnitude of the rise in antibody titer varies considerably between horses, with two horses showing no elevation in antibody levels following booster vaccination. The presence of poor responders poses a risk of infection and clinical disease for these horses, as demonstrated by several case reports of clinical rabies in vaccinated horses [23,24]. In addition to the fatal consequence for these horses, they also pose a potential risk to humans in their surroundings [1]. The rate of poor response to vaccination in horses (as in other animals) should be better evaluated, as it may also breach herd immunity attempted by regional control programs and increase the risk of introduction or emergence of rabies in areas considered free of disease.

In animals, pre-exposure prophylaxis is mainly administered in order to reduce zoonotic risk. However, since horses serve both as companion animals and athletes, animal welfare and economic consequences may also be important. The fact that eight of ten horses showed a considerable increase in antibody titers following booster vaccination may imply that post-exposure prophylaxis (PEP) could be appropriate for horses following an encounter with a rabid animal, especially in pre-vaccinated horses. In humans, PEP is administered in cases of exposure and was shown to efficiently prevent the clinical (and fatal) outcome [2,4,5]. Further research is needed to introduce PEP in horses.

Several factors were found to be associated with antibody response to rabies vaccination in horses, humans and pets. These include the individual’s age, sex, breed, the vaccine type and the number of previous vaccinations [6,7,8,14,15,21,22,25]. In the current study, although age, sex and breed were found to be associated with RVNA levels in the univariable analysis, only the breed remained significant in the multivariable model, demonstrating lower antibody titers in purebred horses than in mixed-breed horses. Nonetheless, none of the examined factors were significantly associated with having an antibody titer below 0.5 IU/mL. 

The regime for primary vaccination is crucial to achieve adequate and prolonged protection. In humans, pre-exposure vaccination includes a series of three doses (at days 0, 7 and 21/28), with a booster after one year required, to ensure immune protection and prolonged immunity. The antibody response to vaccination was significantly higher after this three-dose regime than in cases that initially received only two doses [4,6,7,26]. In animals, the accepted protocol for vaccination only includes the primary administration of a single dose, with a booster after one year [13,27]. However, the difference between different priming regimes in horses has never been investigated.

All horses included in this study were vaccinated with the same vaccine type, since only one brand of vaccine is available for veterinary use in the country. However, one of the limitations of this study was the lack of complete vaccination history. The number of previous vaccinations may affect the magnitude and duration of antibody response, as shown in humans and in dogs [6,7,25]. Therefore, it is possible that horses that had been vaccinated numerous times in the past would have higher antibody titers than horses that had only been vaccinated once or twice in the past. One of the reasons that only horses over two years of age were included in this study was to reduce the likelihood of documenting a primary response in any of the horses. Another limitation of the study, being a retrospective study, was the difficulty in recruiting enough horses that had not been vaccinated for prolonged periods of time but still had accurate information regarding their last vaccination date. Most farms with good management, veterinary care and record keeping usually administer annual booster vaccination, as recommended by most veterinarians. Hence, a larger sample size was available for the 6–24-month interval, while only 10 horses in one farm represent the 91-month timepoint. A future, larger, prospective study is warranted to corroborate the findings of this primary survey.

## 5. Conclusions

This is the first report demonstrating prolonged persistence of protective immunity in horses for three to eight years following rabies vaccination. These primary results should lead to further prospective studies evaluating long-term response to vaccination in horses in order to design an optimal vaccination strategy, with possible prolonged inter-booster intervals. Moreover, the protocol for the primary vaccination regime should also be evaluated in order to ensure the persistence of immunity. In addition, the existence of poor responders to vaccination should be considered in an epidemiological perspective in order to minimize the risk of infection and clinical disease in horses and its zoonotic risk. 

## Figures and Tables

**Table 1:** The range and distribution of rabies-virus-neutralizing antibody levels (RVNA) in horses at different time intervals following vaccination. The number and proportion of horses that had RVNA titer below the level considered protective against infection (0.5 IU/mL) are stated and compared between groups using Fisher’s exact test.

Time Since Vaccination (Years)	N	Range RVNA (IU/mL)	Mean RVNA (IU/mL)	SDV RVNA (IU/mL)	N (%) RVNA < 0.5 IU/mL	Sig Fisher’s Exact
0–2	76	0.03–104.14	10.59	15.11	7 (9.2%)	ref
2–4	21	0.2–41.68	5.81	8.86	2 (9.5%)	1
4–6	19	0.03–43	9.99	10.55	2 (10.5%)	1
6–8	10	0.21–25.84	6.57	8.15	3 (30%)	0.88

**Table 2:** Potential risk factors and their association with rabies-virus-neutralizing antibody levels (RVNA) in 76 horses vaccinated within two years prior to sampling. Mean and standard deviation (SDV) of RVNA are stated for each category, as well as the statistical significance (Sig) of the univariable Kruskal–Wallis (KW) and multivariable generalized estimating equation (GEE) models.

		N	Mean RVNA (IU/mL)	SDV RVNA (IU/mL)	Sig KW ^1^	Sig GEE ^2^
Breed	Mixed	35	15.10	18.86	<0.001	ref
	Tennessee walking horse	20	4.90	10.75		0.012
	Quarter horse	8	10.57	9.75		0.187
	Arabian	6	12.24	8.40		0.663
	Warmblood	6	2.74	1.76		<0.001
	Miniature	1	3.70	na		0.035
Sex	Mare	39	8.96	12.09	0.012	ref
	Gelding	33	13.57	18.41		0.652
	Stallion	4	1.89	2.17		0.631
Housing	Stall	27	13.36	20.68	0.323	
	Paddock	44	8.49	9.59		

^1^ KW—Kruskal–Wallis nonparametric test; ^2^ GEE—generalized estimating equation; na—nonapplicable.

**Table 3:** The times and mean rabies-virus-neutralizing antibody levels (RVNA) are described for 33 horses which were sampled twice at different times following vaccination. The time difference between sampling and the mean difference in RVNA levels are specified.

	N	Time 1	Mean RVNA (IU/mL)	Time 2	Mean RVNA (IU/mL)	Time Difference	Mean RVNA Difference (IU/mL)
Farm 1	4	12 m	17.68	36 m	16.13	24 m	−1.55
Farm 2	4	22 m	14.78	50 m	11.79	28 m	−2.99
Farm 3	11	66 m	9.79	91 m	6.11	25 m	−3.68
Farm 4	14	24 m	1.65	40 m	2.71	16 m	1.06

**Table 4:** The characteristics and rabies-virus-neutralizing antibody levels (RVNA) of ten individual horses immediately before and a week after rabies vaccination booster.

Horse	Farm	Breed	Sex	Age	RVNA before (IU/mL)	RVNA after (IU/mL)	RVNA Difference (IU/mL)
Y1	Farm 5	Quarter horse	Gelding	15	4.82	43.35	38.53
Y2	Farm 5	Mixed	Gelding	9	14	134.77	120.77
Y3	Farm 5	Mixed	Gelding	8	5	134.78	129.78
Y4	Farm 5	Mixed	Gelding	11	6	134.39	128.39
Y6	Farm 5	Paint horse	Mare	16	1.3	1.3	0
Y7	Farm 5	Quarter horse	Mare	7	5	4.32	−0.68
EH2	Farm 6	Mixed	Gelding	9	12.7	28.08	15.38
EH4	Farm 6	Mixed	Gelding	4	4.23	5.4	1.17
EH5	Farm 6	Warmblood	Mare	3	3.92	17.09	13.17
EH6	Farm 6	Warmblood	Mare	13	3.11	5.4	2.29

## Data Availability

All relevant data are included in the manuscript.
